# Successful Management of the Fetal Severe Anemia Associated with Jra Alloimmunization by Intrauterine Transfusion of Jr(a+) Red Blood Cells

**DOI:** 10.1155/2019/5174989

**Published:** 2019-02-24

**Authors:** Masatake Toshimitsu, Shinichi Nagaoka, Shuusaku Kobori, Yuichiro Takahashi, Jun Murotsuki

**Affiliations:** ^1^Department of Maternal and Fetal Medicine, Miyagi Children's Hospital, Miyagi 989-3126, Japan; ^2^Department of Fetal and Maternal Medicine, Nagara Medical Center, Gifu, Japan

## Abstract

**Objective:**

We present a case of fetal severe anemia associated with Jra alloimmunization, which was managed using Doppler measurement of the peak systolic velocity of the fetal middle cerebral artery (MCA-PSV) and intrauterine transfusion (IUT) of Jr(a+) red blood cells (RBCs). We also review the previous case reports on fetal or neonatal anemia associated with Jra alloimmunization.

**Case Report:**

A woman with Jra alloimmunization was referred to our department at 29 weeks of gestation. As fetal MCA-PSV exceeded 1.55 multiples of the median, fetal blood sampling was performed and demonstrated severe anemia. During the course, a total of two IUTs were performed using Jr(a+) RBCs. The neonate was delivered by repeated cesarean section at 35 weeks of gestation and showed no apparent signs of hemolysis.

**Conclusion:**

Based on the literature review, fetal anemia associated with Jra alloimmunization becomes severe during mid-gestation and may not develop during late gestation. The severity of fetal anemia is predicted by MCA-PSV Doppler assessment rather than the maternal anti-Jra titers. Timely IUT of Jr(a+) RBCs can help to prolong the pregnancy to term in emergency situations wherein compatible blood of Jr(a-) RBCs is not available soon.

## 1. Introduction

Fetal anemia can cause fetal high-output cardiac failure, fetal hydrops, iatrogenic preterm delivery, and fetal demise [1]. Therefore, fetal anemia is still an important cause of fetal and neonatal mortality and morbidity in modern obstetric practice.

The Jra antigen, which is a high-incidence red blood cell (RBC) antigen, is known to be involved in hemolytic anemia of the fetus and newborn [2, 3]. The expression of Jra antigen is regulated by the* ATP-binding cassette G2* (*ABCG2*) gene on chromosome 4q22.1 and Jra antigen is located on ABCG2 transporter, which is highly expressed on cells of the erythroid lineage [3, 4]. The Jr(a-) phenotype results from the inheritance of ABCG2 null alleles caused by frameshift or nonsense mutations such as c.1515delC (p.Ala505fs), c.376C>T (p.Gln126Ter), c.421C>A (p.Gln14Lys), and c.1723C>T (p.Arg575Ter), particularly in Japanese individuals [3, 5, 6]. Furthermore, the variation in the Jra antigen density on RBCs among Jr(a+) individuals with genetic mutations at 376 and 421 has also been reported [7].

Although anti-Jra can be produced in Jr(a-) women due to pregnancy or incompatible Jr(a+) RBC transfusion and cross the placenta, the effect of anti-Jra on fetal anemia remains unclear [4, 7–11]. For example, the fetal anemia associated with anti-Jra positivity is reported to be mild [8, 9]. In contrast, several reports have shown an association between Jra alloimmunization and fetal or neonatal severe anemia requiring intervention [4, 10, 11]. In addition, in one case, neonatal death occurred due to Jra alloimmunization [10].

We report a case of fetal severe anemia associated with Jra alloimmunization that was successfully managed by close fetal monitoring and intrauterine transfusion (IUT) of Jr(a+) RBCs. We also analyzed the clinical characteristics and perinatal outcome of fetal anemia due to Jra alloimmunization. Cases were obtained by searching PubMed or Medical Online for the terms “Jr(a)”, “hemolytic disease”, “fetus”, and “anemia”. All reports and publications in English or Japanese from 2006 to 2016 were reviewed. Cases at Nagara Medical Center, a tertiary center in Japan, were also included.

## 2. Clinical Case

The patient was a 34-year-old Japanese woman (gravida 5 para 2, including 2 miscarriages) with blood group O type RhD (+). She was referred to our department in the 29th week of her fifth pregnancy for perinatal management. She had no relevant medical history and had never received a blood transfusion. Screening performed when she was pregnant with her first child revealed Jr(a-) and anti-Jra with a titer of 1:512. Her Jr(a-) genotype was c.376T/T and c.421C/C. Her partner was Jr(a+) with c.376C/C and c.421C/A. Her first child of 2590 g was delivered at 36 weeks of gestation by cesarean section (CS) at a different hospital due to breech presentation and did not have any other episodes of anemia or jaundice. When pregnant with her second child, the titer of anti-Jra changed from 1:128 to 1:256. Her second child was delivered at 37 weeks and 6 days of gestation by planned repeat CS at a different hospital due to previous CS. Her second child of 2808 g was diagnosed with anemia (Hb 8.4 g/dL) based on a positive direct antiglobulin test (DAT), but did not display jaundice. The second child was diagnosed as being heterozygous for c.376C/T with c.421C/C and exhibited the Jr(a+) phenotype.

**Figure 1:**
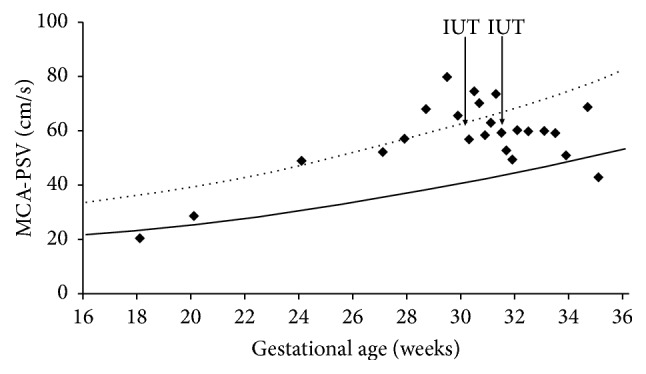
*The peak systolic flow velocity of the fetal middle cerebral artery during pregnancy*. (—) The median MCA-PSV level in normal pregnancies; (^*▪▪*^) 1.55 MoM; (^*◆*^) our case. MCA-PSV: the peak systolic flow velocity of the fetal middle cerebral artery; MoM: multiple of the median.

Her current pregnancy was a naturally conceived and normal singleton pregnancy. The titer of anti-Jra at 20 weeks of gestation was 1:256. No other antibodies against blood group antigens were identified. The fetus was monitored by ultrasound scans and Doppler measurements of the peak systolic flow velocity of the fetal middle cerebral artery (MCA-PSV). Although the titer of anti-Jra did not change (1:256), the MCA-PSV level began to increase at 24 weeks and exceeded 1.55 multiples of the median (MoM) at 28 weeks (Figure 1). She was referred to our department at 29 weeks and 6 days due to suspected fetal anemia. Our ultrasound examination showed that the estimated fetal body weight corresponded to the Japanese standard for the gestational age and that there were no fetal or placental structural abnormalities. The MCA-PSV level (65.6 cm/s) was >1.55 MoM (Figure 1). The fetal cardiothoracic area ratio (CTAR) was 42.0% without any signs of hydrops, such as ascites or skin edema. The next day, percutaneous umbilical cord blood sampling (PUBS) was carried out and revealed fetal severe anemia (Hb 3.5 g/dL, Hct 9.9%) (Table 1). An immediate IUT via the umbilical cord was performed with group O RhD (-), Jr(a+) concentrated RBC units. After the first IUT, the Hb and Hct levels increased to 7.2 g/dL and 22.1%, respectively (Table 1). On the sixth day after the first IUT (30 weeks and 6 days), the titer of anti-Jra increased from 1:256 to 1:512. The IgG subclass was found to be IgG1 and IgG3. On the seventh day after the first IUT, the MCA-PSV level was >1.55 MoM, suggesting the exacerbation of fetal anemia (Figure 1). Thus, a second PUBS and a second IUT was performed with group O RhD (-), Jr(a+) concentrated RBC units on the ninth day after the first IUT. The fetal Hb and Hct levels before the second IUT were 6.1 g/dL and 18.6%, respectively (Table 1), while those after the second IUT were 9.5 g/dL and 29.1%, respectively (Table 1). After the second IUT, an ultrasound examination showed that the MCA-PSV level remained within the normal range and that the CTAR level had normalized to 31.6% (Figure 1). She returned to her referring hospital, where she was managed from 32 weeks of gestation. At 34 weeks of gestation, the fetal MCA-PSV and CTAR levels increased to <1.5 MoM and 39%, respectively (Figure 1). The exacerbation of fetal anemia was suspected; thus, a repeat CS was performed at 35 weeks and 1 day of gestation due to previous CS at the referring hospital. The neonate of 2114 g had anemia (Hb 9.2 g/dL, Hct 28.4%) with a positive DAT, without jaundice (total bilirubin 1.7 mg/dL) (Table 1). On the second day after birth, the neonatal anemia worsened (8.0 g/dL) and a blood transfusion was performed. Neither neonatal anemia nor jaundice developed after the single blood transfusion. The neonate was discharged from the hospital without phototherapy.

To investigate the phenotype and the Jra antigen density of the neonate, an analysis of the* ABCG2 *gene was performed at Japanese Red Cross Tohoku Block Center. Genomic DNA was extracted from the peripheral blood and PCR-SSP was used to examine the genetic base substitutions at positions 376, 421, 1515, and 1723, which are most common in Japanese Jr(a-) individuals [7]. The neonate's phenotype was classified as Jr(a+); the neonate was heterozygous for c.376C/T with no mutation at position 421 (c.421C/C), which was the same genotype of the second child.

## 3. Discussion

We presented a case of fetal severe anemia associated with Jra alloimmunization in which the pregnancy was successfully prolonged by Doppler measurements of the MCA-PSV and timely IUT. This is also the first case report describing the performance of IUT using Jr(a+) concentrated RBC units. In the present case, fetal anemia did not develop after the IUT of Jr(a+) RBCs and the neonate's blood exam did not show any evidence of hemolysis.

Several clinical investigations and experimental studies have been performed to elucidate the precise pathogenesis of fetal anemia associated with Jra alloimmunization [4, 7, 9–17]. There are ten reports, including our own, describing fetal or neonatal anemia in association with Jra alloimmunization (Table 2). A review of the literature revealed several features of the clinical course of fetal anemia with Jra alloimmunization (Table 2). First, the cases that required IUT suggested that the fetal anemia becomes severe during mid-gestation; however, it is unclear when anemia occurs. Furthermore, the fetal anemia was not exacerbated during late gestation. In the present case, fetal anemia did not develop after the second IUT of Jr(a+) RBCs (Table 1). Although the exact mechanisms underlying the development of fetal severe anemia during mid-gestation and the transfusion effect of IUT of Jr(a+) RBCs are unknown, they may be related to the change of ABCG2 expression in erythroid lineage cells with advancing gestational ages [4]. A high expression of ABCG2 on erythroid progenitor cells during mid-gestation may lead to a high affinity for anti-Jra, resulting in development of fetal anemia during mid-gestation [4]. In contrast, a low expression of ABCG2 on RBCs after late gestation may contribute to prevention of the exacerbation of fetal anemia and preservation of efficacy of incompatible transfusion using adult Jr(a+) RBCs [4]. Thus, a single IUT may be sufficient to allow the pregnancy to progress until term without repeated IUTs. However, cases treated with IUT may still require neonatal blood transfusion. Second, the severity of fetal anemia is not directly correlated with the titer of anti-Jra, suggesting that the measurement of the maternal antibody titers alone is not a reliable method for predicting fetal anemia. Future studies with larger sample size focused on the value of the first titer or repeated titer determination in the management of Jra alloimmunization requiring frequent monitoring to detect fetal anemia are helpful to identify the fetus with need for IUT. In the present case, PUBS was indicated based on the measurement of the fetal MCA-PSV. In the majority of cases that were treated with IUT, the timing of the first PUBS was based on the assessment of the fetal MCA-PSV. Although there are no clear data regarding the optimal intervals between repeated Doppler measurements of the fetal MCA-PSV, measurement of the MCA-PSV may be useful in the management of Jra alloimmunization. Third, anti-Jra-mediated fetal anemia was characterized by anemia without developing hyperbilirubinemia at PUBS and at birth. However, there are several limitations. First, the unconjugated fetal bilirubin crosses the placenta. In the present case, we did not evaluate the level of total bilirubin in PUBS at 30 weeks of gestation due to the limited blood sample volume (Table 1). Second, the fetal anemia was not exacerbated after late gestation based on the literature review, including this one (Table 2). Therefore, we cannot assess the contribution of hemolysis to fetal anemia based on the bilirubin levels.

In this review, the majority of clinical data analyzed are derived from Japanese women (9 women were Japanese and 1 was Caucasian of Gypsy Spanish origin [10]), so it is necessary to be cautious about applying our results to other racial groups.

Experimental studies have demonstrated that the ABCG2 is expressed on not only cells of the erythroid lineage but also the placenta and that it is a plasma membrane protein extruding endogenous and exogenous substrates, such as xenobiotics, heme, porphyrin, and uric acid [15–17]. The maintenance of cellular porphyrin and heme homeostasis, which constitute an essential component of hemoglobin, by ABCG2 is thought to perform a vital physiological function in erythropoiesis [17]. Taken together, these clinical and experimental findings suggest that anti-Jra antibodies may directly affect the function of ABCG2 and may cause fetal anemia due to ineffective erythropoiesis.

One of the important findings in our case was that pregnancy could be prolonged by incompatible IUT with Jr(a+) RBC units. In the present case, we were not able to obtain Jr(a-) RBC units because of the low incidence of Jr(a-). As a result, a total of 105 ml of Jr(a+) RBCs were transfused and fetal anemia did not develop after the second IUT. The sufficient efficacy of incompatible transfusion using adult Jr(a+) RBCs may be partly due to a low expression of ABCG2 on adult RBCs compared with fetus [4]. In the setting of incompatible transfusions in adults, whereas most reported cases showed a mild and delayed hemolytic transfusion reaction, an acute hemolytic transfusion reaction also has been reported, suggesting that anti-Jra is a possible cause of transfusion-related hemolysis [18–20]. Therefore, IUT of Jr(a+) RBCs may be considered, especially in emergency situations wherein compatible blood of Jr(a-) RBCs is not available soon [20].

Another important finding in our case was the different outcomes between the second child who did not require IUT and the present child who required IUT. Since the severity of fetal anemia may be independent of the maternal anti-Jra, we hypothesized that the Jra antigen density on fetal RBCs contributes to the severity of anemia. In contrast to our hypothesis, the Jr(a+) genotype was the same in both children, suggesting that their Jra antigen density was similar and implicating the impact of ABCG2 on erythropoiesis and compensatory mechanisms when ABCG2 is inhibited by anti-Jra. There are several possibilities that could explain the intrafamilial phenotypic variability in the perinatal outcomes. One possibility is that increasing sensitization due to a previous Jra positive child may be involved in the development of fetal severe anemia. It is known that the degree of fetal anemia due to red cell alloimmunization depends on the number of affected pregnancies. Another possibility is anti-Jra titer variability. Intra- and interlaboratory variability in the titer measurement constitute a major point of concern. In addition, antibody titration, a semiquantitative method to detect the reactivity, may not reflect true biological activity in the Jra alloimmunization. Although our main goal in the management of pregnancies complicated by Jra alloimmunization is to detect fetuses that are at higher risk of developing severe anemia in order to prevent fetal hydrops [21], the clinical course of fetal anemia may be affected by various factors, such as the Jra antigen density of cells of the erythroid lineage and placenta, the maternal antibody titer, and the numbers of affected pregnancies. It is important to recognize that fetal severe anemia can occur, even if a previous child did not develop anemia.

In conclusion, it is necessary to recognize Jra alloimmunization as a possible cause of fetal severe anemia, and that this may occur through ineffective erythropoiesis and hemolysis. We think that careful obstetric management is needed, including routine Doppler measurements of the fetal MCA-PSV to detect anemia, the sampling of fetal blood by PUBS when fetal anemia is detected, and when necessary timely IUT to improve the neonatal outcomes by preventing fetal hydrops and fetal anemia-associated prematurity.

## Figures and Tables

**Table 1:** The fetal blood analysis.

GA Weeks + days	Pre-IUT Hb(g/dL)/ Hct(%)	Post-IUT Hb(g/dL)/ Hct(%)	Volume of IUT (ml)	T-bil (mg/dL)	DAT
30+0 (first IUT)	3.5/ 9.9	7.2/ 22.1	70	N/A	N/A
31+2 (second IUT)	6.1/ 18.6	9.5/ 29.1	35	N/A	N/A
35+1 (at birth)	9.2/ 28.4		-	1.7	+

GA: gestational age; Hb: hemoglobin; Hct: hematocrit; IUT: intrauterine transfusion; T-bil: total bilirubin; DAT: direct antiglobulin test; N/A: not available.

**Table 2:** Theclinical details and outcomes of the reported cases of Jra alloimmunization, including the present case.

Case	GA at first PUBS or IUT (weeks)	MCA-PSV at first PUBS (MoM)	Hb (g/dL)/Hct (%)/T-bil (mg/dL) at first PUBS	Number of IUTs	Type of blood transfusion	GA at birth (weeks)	Interval between the last IUT and birth (weeks)	Hydrops fetalis	Hb (g/dL)/Hct (%)/T-bil (mg/dL) at birth	Maximum anti-Jra titer	Neonatal transfusion	Neonatal outcome
Cases with IUT (n = 6)												

Present case	30	>1.55	3.5/ 9.9/ N/A	2	Jr(a+)	35	5	-	9.2/ 28.4/ 1.7	512	**+**	Alive
Case 2 (unrelated)	29	>1.55	5.3/ 15.7/ 1.4	1	Jr(a-)	37	8	-	9.4/ 29.0/ 1.1	256	-	Alive
Ishihara et al. [11]	30	>1.55	3.5/ 7.9/ 1.0	4	Jr(a-)	35	1	+	7.2/ N/A/ 1.0	512	**+**	Alive
Fujita et al. [4]	30	>1.5	8.5/ 26.8/ N/A	1	Jr(a-)	37	7	-	11.8/ 35.4/ 2.0	512	-	Alive
Aikou et al. [12]	30	>1.5	5.4/ 15.7/ 2.3	1	Jr(a-)	38	8	-	10.8/ 31.8/ 2.1	32	-	Alive
Yahara et al. [13]	27	N/A	3.4/ 11.4/ 1.9	2	Jr(a-)	37	6	+	12.2/ 37.0/ 1.0	16	-	Alive

Cases without IUT (n = 4)												

Sasamoto et al. [14]	-	-	-	0	-	33	-	-	8.2/ 25.4/ 0.9	1024	-	Alive
Peyrard et al. [10]	-	-	-	0	-	36	-	**+**	6.4/ N/A/ 2.5	1024	**+**	Neonatal death
Endo et al. [7]	-	-	-	0	-	37	-	-	8.4/ 25.8/ 1.9	256	-	Alive
Masumoto et al. [9]	-	-	-	0	-	37	-	-	11.5/ N/A/ N/A	64	-	Alive

GA: gestational age; Hb: hemoglobin; Hct: hematocrit; PUBS: percutaneous umbilical cord blood sampling; IUT: intrauterine transfusion; T-bil: total bilirubin; N/A: not available. Case 2 is unrelated to ours. The clinical data of case 2 was provided by the Department of Fetal-Maternal Medicine, Nagara Medical Center.
